# Role of High Serum Tenascin C Levels as Potential Biomarker of Persistent Inflammation in Patients with Ankylosing Spondylitis Despite Treatment with cs-DMARDS or Anti-TNF Agents

**DOI:** 10.3390/diagnostics15121457

**Published:** 2025-06-07

**Authors:** Alejandro Martelli-Garcia, Yussef Esparza-Guerrero, Heriberto Jacobo-Cuevas, Ana Miriam Saldaña-Cruz, Norma Guadalupe Gonzalez-Montoya, Cesar Arturo Nava-Valdivia, Eli Efrain Gomez-Ramirez, Maria Luisa Vazquez-Villegas, Juan Manuel Ponce-Guarneros, Melissa Ramirez-Villafaña, Norma Alejandra Rodriguez-Jimenez, Alberto Daniel Rocha-Muñoz, Ernesto German Cardona-Muñoz, Jaime Morales-Romero, Laura Gonzalez-Lopez, Jorge Ivan Gamez-Nava

**Affiliations:** 1 Programa de Doctorado en Farmacología, Centro Universitario de Ciencias de la Salud (CUCS), Universidad de Guadalajara, Guadalajara 44340, Jalisco, Mexico; dr.martelligdl@gmail.com; 2 Instituto de Terapéutica Experimental y Clínica, Departamento de Fisiología, Centro Universitario de Ciencias de la Salud (CUCS), Universidad de Guadalajara, Guadalajara 44340, Jalisco, Mexico; esparzaguerreroy@gmail.com (Y.E.-G.); ana.saldanac@academicos.udg.mx (A.M.S.-C.); dr.efrain.gomez@gmail.com (E.E.G.-R.); drponce85@gmail.com (J.M.P.-G.); assilem298910@hotmail.com (M.R.-V.); norma.rodriguezj@academicos.udg.mx (N.A.R.-J.); cameg1@gmail.com (E.G.C.-M.); 3 Departamento de Aparatos y Sistemas II, Decanato de Ciencias de la Salud, Universidad Autónoma de Guadalajara, Av. Patria 1201, Lomas del Valle, Zapopan 45129, Jalisco, Mexico; 4 Group for the Assessment of Prognosis Biomarkers in Autoimmune Disorders, Centro Universitario de Ciencias de la Salud (CUCS), Universidad de Guadalajara, Guadalajara 44340, Jalisco, Mexico; heriberto.jcuevas@alumnos.udg.mx; 5 Programa de Postdoctorado, Departamento de Psicología Básica, Centro Universitario de Ciencias de la Salud (CUCS), Universidad de Guadalajara, Guadalajara 44340, Jalisco, Mexico; 6 Departamento de Bienestar y Desarrollo Sustentable, Centro Universitario del Norte, Universidad de Guadalajara, Guadalajara 44340, Jalisco, Mexico; norma.montoya@cunorte.udg.mx; 7 Departamento de Microbiologia y Patología, Centro Universitario de Ciencias de la Salud (CUCS), Universidad de Guadalajara, Guadalajara 44340, Jalisco, Mexico; dr.arturonaval@gmail.com; 8 Departamento de Epidemiología, Unidad de Medicina Familiar No. 4, Instituto Mexicano del Seguro Social (IMSS), Guadalajara 44340, Jalisco, Mexico; ma_luisavazquez@hotmail.com; 9 Instituto Regional de Investigación en Salud Pública, Programa de Doctorado y Coordinación del Programa de Doctorado en Salud Pública, Departamento de Salud Pública, Centro Universitario de Ciencias de la Salud (CUCS), Universidad de Guadalajara, Guadalajara 44340, Jalisco, Mexico; 10 Departamento de Salud-Enfermedad Como Proceso Individual, Centro Universitario de Tonalá, Universidad de Guadalajara, Guadalajara 44100, Jalisco, Mexico; medalbertorocha@hotmail.com; 11 Instituto de Salud Pública, Universidad Veracruzana, Xalapa 91190, Veracruz, Mexico; jamorales@uv.mx

**Keywords:** ankylosing spondylitis, tenascin C, BASDAI, disease activity

## Abstract

**Background/Objectives:** Ankylosing spondylitis (AS) is a severe chronic inflammatory rheumatic disease involving the spine, sacroiliacs, and peripheral joints. A lack of therapeutic response leads to severe sequelae. Currently, new markers are being tested to identify patients with poor outcomes. Tenascin C (TNC) is involved in triggering some relevant mechanisms of inflammation. Today, it remains unclear whether TNC levels might be useful as a biomarker of persistent activity. The aim of this study was to evaluate in AS whether serum levels of tenascin C are associated with persistent disease activity despite treatment. **Methods:** We included AS patients who had been treated with conventional synthetic disease-modifying antirheumatic drugs (cs-DMARDS) or anti-TNF agents for at least three months in a cross-sectional study. Response was assessed with the Bath Ankylosing Spondylitis Disease Activity Index (BASDAI); scores ≥ 4 indicate persistent disease activity, while scores < 4 indicate inactive disease. Serum TNC levels, C-reactive protein (CRP) levels, and Erythrocyte Sedimentation Rate (ESR) were determined through the ELISA technique, nephelometry, and the Westergren method, respectively. **Results:** We evaluated 58 patients with AS (62.1% men); of them, 33 (56.9%) had persistent active disease (BASDAI ≥ 4) despite treatment and 25 (43.1%) had inactive disease (BASDAI < 4). The median TNC level was 18.6 ng/mL. BASDAI correlated with TNC levels (rho: 0.528, *p* < 0.001), CRP (0.352, *p* = 0.007), and ESR (0.342, *p* = 0.009). Patients with persistently active AS had higher serum TNC levels than those with inactive AS (35.2 vs. 6 ng/mL, *p* < 0.001). No differences in TNC level were found in patients treated with cs-DMARDS vs. anti-TNF agents. The ROC curve for serum tenascin C in active AS patients had an area under the curve = 0.78 (CI 95%: 0.65–0.91) with optimal serum tenascin C cutoff (>13.85 ng/mL). Sensitivity for detecting active AS was higher with TNC compared to ESR and CRP. **Conclusions:** We suggest that an elevated TNC level may be a useful biomarker of persistent disease activity despite treatment in AS; further studies should investigate the role of TNC levels in predicting the progression of the disease.

## 1. Introduction

Ankylosing spondylitis (AS) (or axial spondyloarthritis) is one of the five most common chronic inflammatory rheumatic diseases seen in rheumatology consultations [1,2,3]. This disease involves the axial skeleton, mainly the spine and sacroiliac joints, as well as peripheral joints and other multiple enthesitis, leading to severe inflammation with disability, development of syndesmophytes, and, in severe cases, fusion of the spine [4,5]. The persistence of active disease despite treatment is a strong predictor of the development of sequelae, deteriorated physical functioning, and impairment in quality of life in AS patients. Therefore, the identification of new markers favoring persistent disease activity is mandatory to achieve better results of treatments. The use of clinical indices is useful to clinically identify disease activity in AS, such as the Bath Ankylosing Spondylitis Disease Activity Index (BASDAI) [6], which allows an assessment of the therapeutic response. With this index, disease activity can go from 0, indicating the lowest disease activity, to 10, indicating the highest disease activity; a score of ≥4 is the cutoff for patients with significant disease activity [7].

One of the major goals in treating AS is to achieve reduced disease activity, thereby minimizing disability and improving quality of life [8]. To achieve this goal, conventional synthetic disease-modifying antirheumatic drugs (cs-DMARDS) can be used, or biologic agents, anti-TNF agents, or anti-IL-17 therapies, which are recommended for patients with a refractory AS, or with indicators of poor prognosis [8]. To date, although several biomarkers have been tested in AS, none of them can properly reflect the risk of persistent disease with chronic inflammation.

The search for new markers of disease activity is still required in ankylosing spondylitis (AS) to increase the accuracy of diagnosis, and also to improve assessment of disease activity, predict prognosis, and guide potential modifications in treatment [9]. C-reactive protein (CRP) and erythrocyte sedimentation rate (ESR) are currently used as biomarkers of disease activity. However, these acute phase reactants have several limitations because there are some subgroups of AS patients who do not present elevated levels of these markers and they may be normal in individuals with active disease [10,11].

Evaluating the role of new molecules involved in the development of inflammation in chronic rheumatic diseases remains a task for the future. Among them, tenascin C (TNC) might be related to chronic inflammatory processes, and certain evidence has been published in animal models as well as in patients with autoimmune diseases such as multiple sclerosis and rheumatoid arthritis [12]. However, the role of TNC in AS is still unknown.

**Figure 1:**
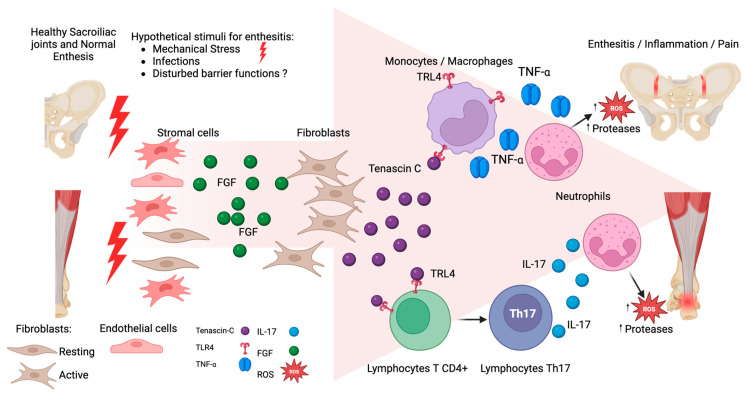
Hypothetical participation of tenascin C (TNC) in enthesitis. At the start, mechanical stress stimulates mesenchymal cell proliferation, mainly fibroblasts, increasing Fibroblast Growth Factor (FGF). The FGF activates the fibroblast production of TNC. TNC binds to TLR4 receptors, promoting the differentiation of T lymphocytes into Th17 cells, leading to an overproduction of IL-17 secretion. Additionally, TNC can activate macrophages and monocytes through binding TLR4, and can release Tumor Necrosis Factor α (TNF-α). Both cytokines IL-17 and TNF-α increase the recruitment of neutrophils, with the release of proteases and reactive oxygen species (ROS) favoring the persistence of inflammation.

TNC is a glycoprotein located in the extracellular matrix of various tissues [13,14]. TNC plays a role in inflammation through activating toll-like receptor 4 (TLR4), triggering the differentiation of naive T cells into Th17 cells [12]. Additionally, TNC activates TLR4 receptors in Th17, neutrophils, macrophages, and dendritic cells and can induce the release of soluble proinflammatory cytokines including interleukin-17 (IL-17), tumor necrosis factor-α (TNF-α) [both key cytokines for the inflammatory process in AS], and other cytokines such as IL-6, IL-8, and IL-1β [9] (see Figure 1) [15,16]. The IL-17 and TNF-α promotes neutrophil recruitment and activates NF-κB signaling, leading to increased reactive oxygen species (ROS), mitochondrial dysfunction, and DNA damage with persistence of the inflammatory process.

Bubova et al. investigated serum TNC levels in patients with axial spondyloarthritis (axSpA) and reported that TNC levels were higher in these patients than in controls, although a significant correlation between TNC levels and disease activity was not demonstrated [17]. In contrast, Gupta et al. reported correlation between serum TNC levels and disease severity assessed with other indices, such as ASDAS-ESR, in a group of AS patients [18].

However, to date, the role of serum TNC levels as a biomarker of persistent disease activity despite treatment in AS patients remains unknown. Therefore, the aim of this study was to evaluate whether serum TNC levels are associated with disease activity in ankylosing spondylitis patients treated with conventional cs-DMARDS or anti-TNF agents.

## 2. Materials and Methods

### 2.1. Study Design

This study was a cross-sectional design.

### 2.2. Patients

The inclusion criteria were as follows: (a) ankylosing spondylitis patients diagnosed by rheumatologists and meeting the 1984 modified New York criteria for ankylosing spondylitis [19], (b) ≥18 years, (c) any gender, and (d) undergoing treatment with conventional synthetic cs-DMARDS (sulfasalazine or methotrexate) or anti-TNF agents at conventional doses for at least three months. The exclusion criteria were the coexistence of autoimmune diseases (such as rheumatoid arthritis or systemic lupus erythematosus), pregnancy, breastfeeding, thyroid disorders, diabetes mellitus, and active infections (including hepatitis C or B virus and HIV), and patients with a history of blood transfusions in the last three months.

### 2.3. Clinical Assessment

Two trained rheumatologists performed a structured clinical history and physical examination to obtain information on (a) sociodemographic variables (age, sex), (b) epidemiological variables, (c) characteristics of the disease, and (d) therapeutic variables (cs-DMARDS, anti-TNF agents, glucocorticoids, and azathioprine). All patients received one of the following therapeutic strategies for at least 3 months before the study’s onset: (a) cs-DMARDS (sulfasalazine: 1 to 3 g/day orally or/and methotrexate: 10 to 15 mg weekly) or (b) anti-TNF agents (subcutaneous etanercept: 50 mg weekly, or subcutaneous adalimumab: 40 mg every other two weeks, or intravenous infliximab: 5 mg/kg at weeks 0, 2, and 6, then every 6–8 weeks thereafter, or subcutaneous golimumab: 50 mg monthly. Some patients treated with anti-TNF agents received cs-DMARDS simultaneously. Patients with AS-associated uveitis were treated with azathioprine: 2–3 mg/kg. Patients receiving nonsteroidal anti-inflammatory drugs (NSAIDs) and glucocorticoids had to be stable ≥4 weeks prior to the study’s onset.

### 2.4. Biochemical Analysis

All venous blood samples were collected after at least 8 h of fasting via venipuncture in the early morning hours. They were placed in tubes without anticoagulant and centrifuged within the first 30 min.

The serum was separated into several aliquots and stored at −80 °C until analysis.

#### 2.4.1. Determination of Acute-Phase Reactants

Serum C-reactive protein (CRP) levels were determined through nephelometry according to the manufacturer’s instructions, and the erythrocyte sedimentation rate (ESR) was measured via the Westergren method. Both analyses were performed on the same day the sample was collected.

#### 2.4.2. Quantification of TNC Levels in the Serum

Serum TNC levels were measured using the sandwich enzyme-linked immunosorbent assay (ELISA) technique with a human TNC ELISA kit (MyBioSource, Vancouver, British, Columbia, CA, USA; catalogue number MBS723481) following the manufacturer’s protocol. The assay has a sensitivity of 1.0 ng/mL, and an Inter-assay and Intra-assay coefficient of variation of 6.9% and 5.7%, respectively.

### 2.5. Main Outcome Variable

#### Assessing Persistence of Disease Activity Despite Treatment

Two trained rheumatologists assessed persistent disease activity using the Bath Ankylosing Spondylitis Disease Activity Index (BASDAI) as described by Garrett et al. in 1994 [6]. Persistence of disease activity was considered when the BASDAI score was 4 or higher despite treatment with cs-DMARDS or anti-TNF agents for at least three months, whereas a BASDAI score ≤3 was considered to indicate inactive disease [7].

### 2.6. Statistical Analysis and Sample Size

Sample size was computed to detect differences between persistent disease activity and patients with therapeutic response. Based on the article by Gupta et al. [18], who reported that levels of serum TNC in AS with therapeutic response fell from 630.8 ng/mL to 376.4 ng/mL, we assumed a difference in the responders to treatment compared with non-responders of 255 ng/mL, with an estimated SD of 300 ng/mL, a power 0.8, and a significance *p*-value of 0.05; we required a minimum of 23 AS non-responders and 23 AS responders to be able to reject the null hypothesis and to demonstrate that TNC levels can differentiate between the two groups. Quantitative variables are presented as medians (ranges), whereas qualitative variables are presented as frequencies (percentages). Correlation analyses of variables were carried out via the Spearman test, and the results are presented as Spearman’s r (rho). Differences in percentage between groups were identified via chi-square tests. Differences in medians were identified by the Mann–Whitney U test. Significance was set at a *p* value of ≤0.05.

### 2.7. Ethical Considerations

This research was approved by the Committee under the registration number R-CEI/498/2019. The study was conducted in compliance with the guidelines of the Declaration of Helsinki. Participation was entirely voluntary, and all the patients provided informed consent.

## 3. Results

Fifty-eight patients were evaluated, with a median age of 51 years and a male predominance of 62.1%. The median disease duration was 10 years, with a range between 1 and 47 years (see Table 1).

Disease activity parameters were evaluated. The median CRP was 7.5 mg/dL, with 62.1% of patients having high CRP levels (≥5 mg/dL). The median ESR was 15 mm/Hr, with 34.5% of patients having high ESRs (≥20 mm/Hr). Disease activity was measured via BASDAI, and the median BASDAI score was 4.52; active AS disease was present in 56.9% of patients and inactive AS disease was present in 43.1% (see Table 1).

Among the treatments used by patients with AS, 87.9% of patients used cs-DMARDS. The most commonly used treatments were sulfasalazine (70.7%), methotrexate (22.4%) and azathioprine (24.1%). On the other hand, 32.8% of patients reported the use of anti-TNF agents, of which the main agent used was etanercept (20.7%). Glucocorticoid use was reported by 22.4% of patients (see Table 1).

Correlation analysis revealed that the TNC level was positively correlated with the CRP level (rho: 0.295, *p* = 0.02) and with the BASDAI score (rho: 0.528, *p* < 0.001). On the other hand, the BASDAI score was correlated with the CRP level, ESR, and TNC level (see Table 2).

**Figure 2:**
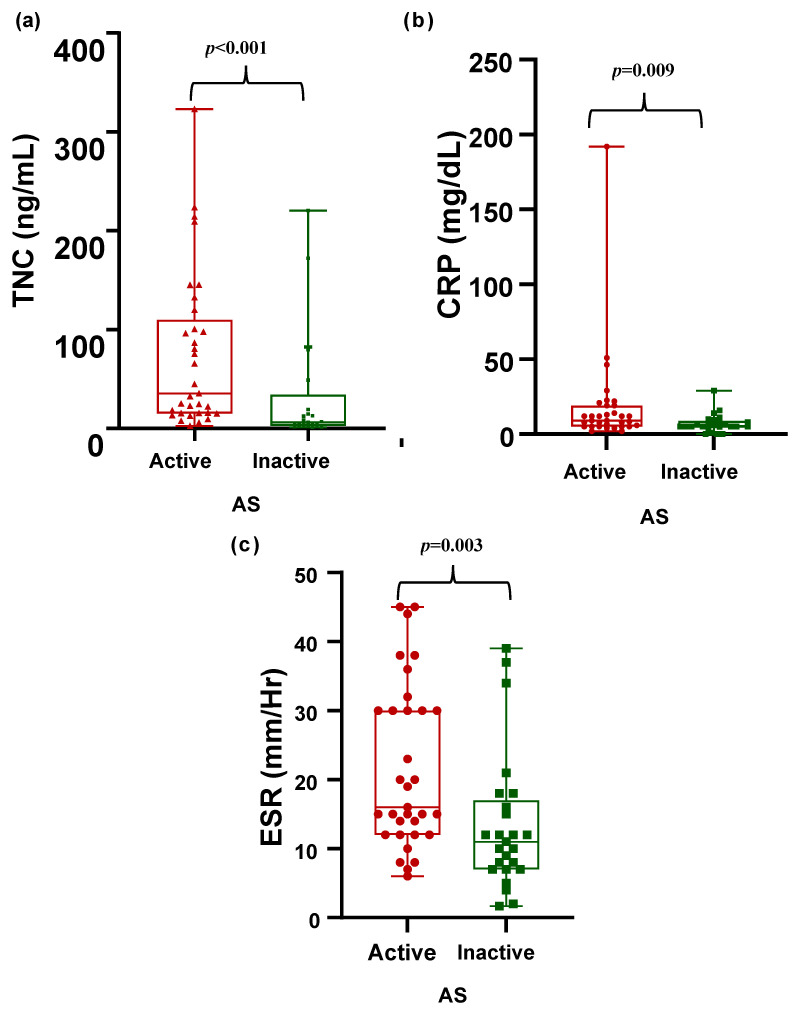
Comparison of disease activity markers in patients with active AS vs. inactive AS. Tenascin C (ng/mL), CRP (mg/dL), and ESR (mm/Hr) levels are shown. (**a**) Comparison of tenascin C levels (ng/mL) between active and inactive AS. (**b**) Comparison of CRP levels (mg/dL) between active and inactive AS. (**c**) Comparison of ESR levels (mm/Hr) between active and inactive AS.

In the comparison of patients with active and inactive AS, patients with active AS had higher CRP levels and ESRs. Patients with active AS had higher TNC levels than those with inactive AS did (35.2 vs. 6 ng/mL, *p* < 0.001) (Table 3, Figure 2).

Table 4 shows a comparison of treatments between patients with active AS vs. inactive AS. No significant differences were found between patients with active and inactive AS in terms of types of treatment. No significant differences were found between AS patients with treatment of cs-DMARDS or Anti-TNF agents (see Table 5).

**Figure 3:**
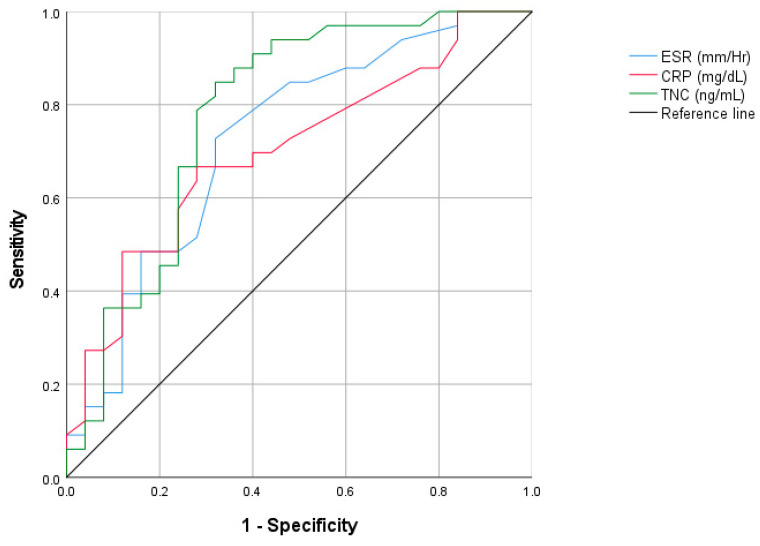
ROC curve analysis comparing ESR, CRP, and tenascinC in identifying active AS.

Figure 3 shows the ROC curve analysis comparing ESR, CRP, and tenascin C in identifying active AS. The ROC curve for serum tenascin C in active AS patients had an area under the curve = 0.78 (CI 95%: 0.65–0.91) with optimal serum tenascin C cutoff (> 13.85 ug/mL), for ESR the area under the curve = 0.72 (CI 95%: 0.59–0.86), and for CRP the area under the curve = 0.70 (CI 95%: 0.56–0.83).

Table 6 shows the utility values for using ESR, CRP, and two cutoffs for TNC. The highest sensitivity for identifying active AS was observed using a TNC cutoff of 13.85 (81.8%), superior to that obtained with ESR or CRP. The highest specificity was observed with ESR (84%), followed by using a cutoff of 13.85 (68%). PPV(+) values were higher with ESR (80%), followed by TNC (77.1%).

In the Supplementary Tables S1 and S2 we performed a logistic regression analysis in order to identify factors associated with active disease (BASDAI ≥ 4). In the first model (Table S1) we included the following as covariables: elevated serum tenascin C (TNC ≥ 13.85 ng/mL); erythrocyte sedimentation rate (ESR ≥ 20 mm/Hr); C-reactive protein (CRP ≥ 5 mg/dL), and adjusted by sex (male), age, and disease duration. After adjustment for potential confounders, only TNC (≥13.85 ng/mL) and ESR (≥20 mm/Hr) increased the risk of active disease. Elevated TNC (OR: 15.57, CI 95%: 3.31–73.19, *p* = 0.001) and elevated ESR (OR: 4.85, CI 95%: 1.09–21.59, *p* = 0.04) were associated with higher risk of active AS, whereas age was associated with lower risk (OR: 0.92, CI 95%: 0.85–0.99, *p* = 0.03). In the second multivariable model (Table S2), we included anti-TNF agents, cs-DMARDs, and glucocorticoids as potential confounders. The risk of high TNC and ESR with active AS persisted significantly independently of the treatments.

## 4. Discussion

This study revealed that patients with ankylosing spondylitis who presented with active disease had higher TNC levels than AS patients with inactive disease. Both groups were treated with anti-TNF agents or cs-DMARDS. Additionally, the TNC level was correlated with the CRP level and BASDAI score. These results contrast with those presented by Gupta et al., who reported a correlation between the TNC level and the erythrocyte sedimentation rate and ERS ASDAS score [18]. The Ankylosing Spondylitis Disease Activity Scale (ASDAS) is another tool designed to evaluate the disease activity level in individuals with ankylosing spondylitis (AS) [20]. Gupta et al. did not find a correlation with the BASDAI score; this may be due to the relatively small sample size, which only included 36 patients with AS treated with NSAIDs exclusively, 75% of whom had active disease according to the BASDAI score [18].

In contrast, our patients with ankylosing spondylitis had been treated with cs-DMARDS (87.9%) and/or anti-TNF agents (32.8%) for at least three months.

In another study, Bubova et al. compared the serum TNC levels of a group of healthy controls vs. patients with spondylarthritis, 45 of whom had AS (Bubová et al., 2020) [17]; they found higher levels of TNC in AS patients. However, 60% of AS patients were treated with NSAIDs, 31.1% with biologic agents, and only 11.1% with cs-DMARDS. Bubova et al. [17] reported no difference in TNC levels among patients treated with NSAIDs compared to cs-DMARDS or anti-TNF agents.

TNC is a large hexameric protein of the extracellular matrix that is poorly expressed in healthy tissues, but the expression of this protein is upregulated in response to tissue damage [12]. TNC can have proinflammatory effects by interacting with toll-like receptor 4 (TLR4), triggering the production of various proinflammatory cytokines, including IL-6, IL-8, TNF-α, and IL-17 [12,21,22].

The causes of TNC overexpression include cancer, cardiovascular diseases, and other chronic disorders [12,23]. Some studies have shown that TNC expression is upregulated in patients with cardiovascular diseases [24,25]. In patients with coronary disease, serum TNC levels were significantly higher (15.51 ± 7.59 ng/mL) compared to healthy controls (*p* < 0.001) [26]. TNC levels are elevated in patients with osteoarthritis, rheumatoid arthritis, and systemic lupus erythematosus, among other diseases, compared with controls [23,27,28]. In inflammatory bowel disease, TNC expression is significantly increased in both intestinal mucosa and serum, correlating with histological inflammation and immune cell infiltration [27].

Page et al. reported higher TNC levels in patients with inflammatory articular disorders compared to controls [29]. They reported a median serum tenascin C level of 118.2 ng/mL in rheumatoid arthritis and 53.8 ng/mL in AS [29]. However, serum TNC levels in patients with AS have been investigated in only a few studies [14,15]. In ligament tissue samples from AS patients and animal models of spondyloarthropathies, TNC levels have been shown to be associated with enthesopathy, promoting pathological bone formation and leading to syndesmophytes [18,19]. Therefore, it is important to continue studying the role of TNC in the generation of inflammation, as well as its potential usefulness as a biomarker, in AS patients. The role of TNC as a potential marker of disease activity in ankylosing spondylitis is still unknown. Established markers such as CRP and ESR have been used for decades in AS. However, both CRP and ESR have limited sensitivity and specificity and can be observed as normal values in a subgroup of patients with inflammation [30,31]. Previous studies have reported that high values of tenascin C are associated with disease activity in AS. Gupta et al. observed a correlation of serum tenascin C with ESR and ASDAS-ESR (r = 0.367, *p* = 0.028) [18]. Similarly, Al-Hindawi et al. identified a correlation between serum TNC and ESR (r = 0.27, *p* = 0.02) in a group of patients with axial spondyloarthritis (axSpo) [32]. Conversely, Buvova et al. did not observe correlations between TNC and BASDAI or CRP. Nevertheless, to date, no studies have compared the potential role of TNC levels with ESR and CRP as a clinical biomarker of disease activity [17]. Using ROC curves, we analyzed the most efficient cutoff of TNC levels to identify active disease (BASDAI ≥ 4) in AS. Using a cutoff ≥13.85 µg/mL (AUC = 0.78), we obtained a higher sensitivity with TNC levels (81.8%) than CRP (72.7%) or ESR (48.5%) to discriminate between active and inactive disease. The positive predictive value of TNC to identify patients with active disease was 77%, very similar to CRP and ESR [17]. Since CRP and ESR cannot identify a subgroup of patients with active AS, we propose that serum TNC can be included in the armamentarium used by clinicians for making clinical decisions in the treatment of this disease.

Our study has several limitations that should be considered in future studies. The first limitation is related to the cross-sectional design, as we captured only a snapshot of the relationship between TNC levels and disease activity severity at a single point in time. This design did not allow us to determine whether these elevated TNC levels existed before the onset of inflammation. Another limitation is that changes in TNC serum levels, and whether other factors influence disease activity and TNC levels, could not be analyzed because of the cross-sectional design of the study. Therefore, longitudinal studies with controls for the type of treatments used are needed to explore the relationship between therapeutic response and changes in TNC levels. Finally, we excluded endocrinopathies such as diabetes mellitus and thyroid diseases from this study in order to avoid the potential effect on TNC levels; therefore, this exclusion could limit the external validity of our results.

Among the strengths of this work are that this study is one of the first studies in which TNC levels were examined in patients with AS treated with cs-DMARDS and/or anti-TNF drugs, and that serum TNC levels were evaluated and compared in AS patients with active vs. inactive disease. Based on our results, TNC level measurement may be of value for the determination of disease activity in AS. Further studies should evaluate whether TNC levels could be useful as predictors of therapeutic response or other outcomes including the radiographic progression.

## 5. Conclusions

In conclusion, we demonstrated that elevated TNC levels were correlated with persistent activity assessed by BASDAI and CRP levels in ankylosing spondylitis. High TNC levels are associated with inflammation independently of treatment with cs-DMARDS or anti-TNF-agents. We suggest that an elevated TNC level may be a useful biomarker of persistent disease activity despite treatment in AS. We propose that the measurement of TNC levels can be considered a complement of CRP and ESR as a tool for identifying active disease, particularly in the subgroup of patients with high BASDAI scores who have normal values of the acute phase reactants; further studies should investigate the role of TNC levels in predicting the progression of the disease.

## Figures and Tables

**Table 1:** Clinical characteristics and therapeutic variables of patients with ankylosing spondylitis.

Variable	AS
	(n = 58)
Age in years, median (range)	51 (19–73)
Male, n (%)	36 (62.1)
Disease duration in years, median (range)	10 (1–47)
CRP, median (range)	7.5 (0–192.0)
CRP Positive, ≥5 mg/dL, n (%)	36 (62.1)
ESR, median (range)	15 (1.70–45)
ESR positive, ≥20 mm/Hr, n (%)	20 (34.5)
BASDAI, median (range)	4.52 (1.1–9.2)
Active AS, BASDAI ≥ 4 n (%)	33 (56.9)
Inactive AS, BASDAI < 4 n (%)	25 (43.1)
Tenascin C, ng/mL median (range)	18.6 (1.2–323.4)
NSAIDs, n (%)	58 (100)
cs-DMARDS, n (%)	51 (87.9)
- Sulfasalazine, n (%)	41 (70.7)
- Methotrexate, n (%)	13 (22.4)
Anti-TNF agents, n (%)	19 (32.8)
- Etanercept, n (%)	12 (20.7)
- Adalimumab, n (%)	5 (8.6)
Glucocorticoids, n (%)	13 (22.4)
Azathioprine, n (%)	14 (24.1)

AS: Ankylosing spondylitis, BASDAI: Bath Ankylosing Spondylitis Disease Activity Index, TNC: Tenascin C, ESR: Erythrocyte Sedimentation Rate, CRP: C-reactive protein. NSAIDs: Non-steroidal anti-inflammatory drugs, cs-DMARDS: Conventional synthetic disease-modifying anti-rheumatic drugs. Quantitative variables are expressed in median and range. Qualitative variables are expressed in frequency and percentage.

**Table 2:** Correlation between tenascin C (TNC) levels and BASDAI with clinical characteristics in AS patients.

Characteristics	TNC Levels		BASDAI Score	
	rho	*p*-Value	rho	*p*-Value
Age (years)	−0.245	0.06	0.118	0.38
Disease duration (years)	0.002	0.99	0.197	0.14
CRP (mg/dL)	0.295	0.02	0.352	0.007
ESR (mm/hr)	0.203	0.12	0.342	0.009
BASDAI score	0.528	<0.001	-	-
Tenascin C levels (ng/mL)	-	-	0.528	<0.001

BASDAI: Bath Ankylosing Spondylitis Disease Activity Index, TNC: Tenascin-C, ESR: Erythrocyte Sedimentation Rate, CRP: C-reactive protein. All the correlations were performed with Spearman’s correlation analysis. Statistical significance was set at *p*: ≤0.05.

**Table 3:** Comparison of clinical and laboratory characteristics between active AS vs. inactive AS.

Variable	Active AS	Inactive AS	*p*-Value
	(n = 33)	(n = 25)	
Age in years, median (range)	51 (26–73)	46 (19–61)	0.3
Male, n (%)	19 (57.6)	17 (68.0)	0.4
Disease duration (yrs), median (range)	15 (2–47)	9 (1–30)	0.1
CRP, median (range)	9 (2.0–192.0)	5 (0.0–29.0)	0.009
CRP Positive, ≥5 mg/dL, n (%)	24 (72.7)	12 (48.0)	0.055
ESR, median (range)	16 (6.0–45.0)	11 (1.7–39.0)	0.003
ESR positive, ≥20 mm/Hr, n (%)	16 (48.5)	4 (16.0)	0.01
Tenascin C, ng/mL, median (range)	35.2 (2.4–323.0)	6 (1.2–220.5)	<0.001

AS: Ankylosing spondylitis, Active AS (BASDAI: Bath Ankylosing Spondylitis Disease Activity Index ≥ 4), Inactive AS (BASDAI < 4), TNC: Tenascin C, ESR: Erythrocyte Sedimentation Rate, CRP: C-reactive protein. Quantitative variables are expressed in medians and ranges, qualitative in frequency and percentage. The chi-square test was used for qualitative variables and the Mann–Whitney U test for quantitative variables. Statistical significance was set at *p*: ≤0.05.

**Table 4:** Comparison of treatments between patients with active AS vs. inactive AS.

Variable	Active AS	Inactive AS	*p*-Value
	(n = 33)	(n = 25)	
cs-DMARDS, n (%)	30 (90.9)	21 (84.0)	0.4
- Sulfasalazine, n (%)	22 (66.7)	19 (76.0)	0.4
- Methotrexate, n (%)	9 (27.3)	4 (16.0)	0.3
Anti-TNF agents, n (%)	9 (27.3)	10 (40.0)	0.3
- Etanercept, n (%)	5 (15.2)	7 (28.0)	0.2
- Adalimumab, n (%)	3 (9.1)	2 (8.0)	1.0
- Other Anti-TNF agents n (%)	1 (3.0) *	1 (4.0) **	1.0
Glucocorticoids, n (%)	8 (24.2)	6 (24)	1.0
Azathioprine, n (%)	9 (27.3)	5 (20.0)	0.5

BASDAI: Bath Ankylosing Spondylitis Disease Activity Index, Active AS (BASDAI ≥ 4), Inactive AS (BASDAI < 4), cs-DMARDS: Conventional synthetic disease-modifying anti-rheumatic drugs, Anti-TNF agents: anti-tumor necrosis factor agents. Other Anti-TNF agents: * Golimumab, ** Infliximab Qualitative variables are expressed in frequency and percentage. The chi-square test was used for qualitative variables. Statistical significance was set at *p*: ≤0.05.

**Table 5:** Comparison of tenascin C (TNC) levels and other clinical characteristics between patients with ankylosing spondylitis treated with anti-TNF agents vs. cs-DMARDS.

Variable	Anti-TNF Agents	cs-DMARDS	*p*-Value
	(n = 19)	(n = 51)	
Age, years, median (range)	45 (19–56)	50 (19–73)	0.060
Male, n (%)	11 (57.9)	32 (62.7)	0.71
Duration of Disease, years, median (range)	9 (1–30)	10 (1–47)	0.56
CRP (mg/dL), median (range)	7.3 (2–51)	7.8 (0–192)	0.91
CRP Positive, ≥5 mg/dL, n (%)	13 (68.4)	23 (59.0)	0.49
ESR (mm/Hr), median (range)	12 (2–45)	15 (2–45)	0.33
ESR positive, ≥20 mm/Hr, n (%)	6 (31.6)	14 (35.9)	0.75
BASDAI Score, median (range)	3.2 (1.4–8.6)	4.8 (1.1–9.2)	0.25
Active AS, BASDAI ≥ 4 n (%)	9 (47.4)	24 (61.5)	0.31
Tenascin C, ng/mL, median (range)	12.4 (1.4–172.3)	21.8 (1.2–323)	0.11

BASDAI: Bath Ankylosing Spondylitis Disease Activity Index, TNC: Tenascin C, ESR: Erythrocyte Sedimentation Rate, CRP: C-reactive protein, DC-ART: disease controlling antirheumatic therapy. Quantitative variables are expressed in median and range, qualitative in frequency and percentage. The chi-square test was used for qualitative variables and the Mann–Whitney U test for quantitative variables. Statistical significance was set at *p*: ≤0.05.

**Table 6:** Utility values of ESR, CRP, and two cutoffs of tenascin C as biomarkers of disease activity in ankylosing spondylitis.

Variable	ESR Cutoff ≥20 mm/Hr	CRP Cutoff ≥5 mg/dL	TNC Cutoff ≥13.85 ng/mL
Sensitivity %, (95% CI)	48.5 (30.8–66.5)	72.7 (54.5–86.7)	81.8 (64.5–93.0)
Specificity %, (95% CI)	84 (63.9–95.5)	52 (31.3–72.2)	68 (46.5–85.1)
PPV(+) %, (95% CI)	80 (60.4–91.3)	66.6 (55.8–75.9)	77.1 (65.1–85.9)
NPV(−) %, (95% CI)	55.26 (45.9–64.2)	59.1 (42.4–73.89)	73.9 (56.7-85.98)
LR(+)	3.03 (1.15–7.95)	1.51 (0.96–2.4)	2.55 (1.4–4.6)
LR(−)	0.61 (0.42–0.89)	0.52 (0.27–1.03)	73.9 (56.7–85.98)
Prevalence	56.9 (43.2–69.8)	56.9 (43.2–73.9)	56.9 (43.2–69.8)

CI: Confidence Interval 95%. PPV(+): Positive predictive value, NPV(−): Negative predictive value, LR(+): Positive Likelihood ratio, LR(−): Negative Likelihood ratio.

## Data Availability

The datasets and statistical analyses generated in this study are available by request to the authors: ivangamezacademicoudg@gmail.com and hjacobocuevas@gmail.com.

## Supplementary Materials

The following supporting information can be downloaded at: https://www.mdpi.com/article/10.3390/diagnostics15121457/s1, Table S1: Model 1. Factors Associated with active AS in multivariable analysis; Table S2: Model 2. Factors Associated with active AS in multivariable analysis.
