# Supplementary material for: Role of High Serum Tenascin C Levels as Potential Biomarker of Persistent Inflammation in Patients with Ankylosing Spondylitis Despite Treatment with cs-DMARDS or Anti-TNF Agents
Source: Diagnostics (Basel). 2025 Jun 7;15(12):1457. doi: 10.3390/diagnostics15121457 (PMC12191457; doi:10.3390/diagnostics15121457)
Supplement: Supplementary file 1 [file diagnostics-15-01457-s001.zip › diagnostics-3645922-supplementary.pdf]

diagnostics MDPI

Supplementary Tables

Suplemmentary Table S1.- Model 1. Factors Associated with active AS in multivariable analysis

	Enter			Stepwise		
**Patients with AS n = 58**	***OR***	**CI 95%**	***p***	***OR***	**CI 95%**	***p***
Male	2.52	0.52– 12.22	0.25			
Age	0.93	0.86 – 1.008	0.08	0.92	0.85 – 0.99	0.03
Disease duration	0.95	0.87 – 1.04	0.28	-	-	-
Elevated TNC, >13.85 ng/mL	16.71	3.40 – 82.05	0.001	15.57	3.31 – 73.19	0.001
ESR ≥20 mm/Hr	4.53	0.75 – 27.46	0.10	4.85	1.09 – 21.59	0.04
CRP≥ 5 mg/L	1.03	0.186 – 5.72	0.97	-	-	-

Logistic regression model: Dependent variable: Active AS (BASDAI ≥4), covariables: Elevated serum Tenascin-C (TNC ≥13.85 ng/mL); Erythrocyte sedimentation rate (ESR ≥20 mmHr); C-Reactive protein (CRP≥ 5 mg/L); Variables for adjusting: sex male, age, disease duration.

Multivariable logistic regression analysis. OR: odds ratio; 95% CI: 95% confidence interval. Crude ORs were obtained using the Enter method. Adjusted ORs were obtained using the Backward Stepwise method.

Suplemmentary Table S2.- Model 2. Factors Associated with active AS in multivariable analysis

	Enter			Stepwise		
**Patients with AS n = 58**	***OR***	**CI 95%**	***p***	***OR***	**CI 95%**	***p***
Male	2.43	0.47– 12.40	0.29			
Age	0.92	0.84 – 1.00	0.06	0.93	0.86 – 0.99	0.04
Disease duration	0.95	0.87 – 1.05	0.29	-	-	-
Elevated TNC, >13.85 ng/mL	27.24	4.27 – 173.85	<0.001	15.57	3.31 – 73.19	0.001
ESR ≥20 mm/Hr	6.84	0.93 – 50.40	0.059	4.85	1.09 – 21.59	0.04
CRP≥ 5 mg/L	1.0	0.147 – 6.86	1.00	-	-	-
Anti-TNF agents	0.51	0.09 – 3.04	0.46	-	-	-
Cs-DMARDS	1.96	0.33 – 11.64	0.46	-	-	-
Glucocorticoids	4.45	0.64 – 31.15	0.13	-	-	-

Logistic regression model: Dependent variable: Active AS (BASDAI ≥4), covariables: Elevated serum Tenascin-C (TNC ≥13.85 ng/mL); Erythrocyte sedimentation rate (ESR ≥20 mmHr); C-Reactive protein (CRP≥ 5 mg/L); Variables for adjusting: sex male, age, disease duration; Anti-TNF agents, Cs-DMARDS and Glucocorticoids

Multivariable logistic regression analysis. OR: odds ratio; 95% CI: 95% confidence interval. Crude ORs were obtained using the Enter method. Adjusted ORs were obtained using the Backward Stepwise method.

References

1. Vanhoof J, Declerck K, Geusens P. Prevalence of rheumatic diseases in a rheumatological outpatient practice. Ann Rheum Dis. 2002 May;61(5):453–5.
2. Miedema HS, van der Linden SM, Rasker JJ, Valkenburg HA. National database of patients visiting rheumatologists in The Netherlands: the standard diagnosis register of rheumatic diseases. A report and preliminary analysis. Rheumatology. 1998;37(5):555–61.
3. Ng X, Low AHL, Chew L, Chong YY, Fong KY, Lui NL, et al. Disease patterns of rheumatology outpatients seen in a tertiary hospital serving a multi-ethnic, urban Asian population in Singapore. Int J Rheum Dis. 2013;16(3):273–8.
4. Murphy SN, Nguyen BA, Singh R, Brown NJ, Shahrestani S, Neal MT, et al. A brief human history of ankylosing spondylitis: A scoping review of pathogenesis, diagnosis, and treatment. Surg Neurol Int. 2022;13:297.
5. Nikiphorou E, Ramiro S. Work Disability in Axial Spondyloarthritis. Curr Rheumatol Rep. 2020;22(9):55.
6. Garrett S, Jenkinson T, Kennedy LG, Whitelock H, Gaisford P, Calin A. A new approach to defining disease status in ankylosing spondylitis: the Bath Ankylosing Spondylitis Disease Activity Index. J Rheumatol. 1994;21(12):2286–91.
7. Ramiro S, Nikiphorou E, Sepriano A, Ortolan A, Webers C, Baraliakos X, et al. ASAS-EULAR recommendations for the management of axial spondyloarthritis: 2022 update. Ann Rheum Dis. 2023;82(1):19–34.
8. Caso F, Costa L, Del Puente A, Di Minno MND, Lupoli G, Scarpa R, et al. Pharmacological treatment of spondyloarthritis: exploring the effectiveness of nonsteroidal anti-inflammatory drugs, traditional disease-modifying antirheumatic drugs and biological therapies. Ther Adv Chronic Dis. 2015;6(6):328–38.
9. Marzeda AM, Midwood KS. Internal Affairs: Tenascin-C as a Clinically Relevant, Endogenous Driver of Innate Immunity. Journal of Histochemistry & Cytochemistry. 2018;66(4):289–304.
10. Tucić M, Stamenković V, Andjus P. The Extracellular Matrix Glycoprotein Tenascin C and Adult Neurogenesis. Front Cell Dev Biol. 2021;9.
11. Chiquet M. Tenascin-C: From Discovery to Structure-Function Relationships. Front Immunol. 2020;11.
12. Ozanne J, Lewis M, Schwenzer A, Kurian D, Brady J, Pritchard D, McLachlan G, Farquharson C, Midwood KS. Extracellular matrix complexity in biomarker studies: a novel assay detecting total serum tenascin-C reveals different distribution to isoform-specific assays. Front Immunol. 2023; 14:1275361.
13. Pas J, Wyszko E, Rolle K, Rychlewski L, Nowak S, Żukiel R, et al. Analysis of structure and function of tenascin-C. Int J Biochem Cell Biol. 2006;38(9):1594–602.
14. Bubová K, Prajzlerová K, Hulejová H, Gregová M, Mintálová K, Hušáková M, et al. Elevated tenascin-C serum levels in patients with axial spondyloarthritis. Physiol Res [Internet]. 2020 Aug 26;653–60. Available from: https://www.biomed.cas.cz/physiolres/pdf/2020/69_653.pdf
15. Gupta L, Bhattacharya S, Aggarwal A. Tenascin-C, a biomarker of disease activity in early ankylosing spondylitis. Clin Rheumatol [Internet]. 2018 May 8;37(5):1401–5. Available from: http://link.springer.com/10.1007/s10067-017-3938-5
16. Linden S Van Der, Valkenburg HA, Cats A. Evaluation of Diagnostic Criteria for Ankylosing Spondylitis. Arthritis Rheum. 1984;27(4):361–8.
17. Lukas C, Landewé R, Sieper J, Dougados M, Davis J, Braun J, et al. Development of an ASAS-endorsed disease activity score (ASDAS) in patients with ankylosing spondylitis. Ann Rheum Dis. 2009;68(1):18–24.
18. Onuora S. Tenascin C promotes pathological bone formation in AS. Nat Rev Rheumatol. 2021;17(7):379–379.

19. Li Z, Chen S, Cui H, Li X, Chen D, Hao W, et al. Tenascin-C-mediated suppression of extracellular matrix adhesion force promotes entheseal new bone formation through activation of Hippo signalling in ankylosing spondylitis. Ann Rheum Dis. 2021;80(7):891–902.
20. Matsui K, Torii S, Hara S, Maruyama K, Arai T, Imanaka-Yoshida K. Tenascin-C in Tissue Repair after Myocardial Infarction in Humans. *International Journal of Molecular Sciences*. 2023; 24(12):10184.
21. Gholipour, A., Shakerian, F., Zahedmehr, A. *et al. Tenascin-C* as a noninvasive biomarker of coronary artery disease. *Mol Biol Rep. 2022;* **49**, 9267–9273.
22. Matsui K, Torii S, Hara S, Maruyama K, Arai T, Imanaka-Yoshida K. Tenascin-C in Tissue Repair after Myocardial Infarction in Humans. *International Journal of Molecular Sciences*. 2023; 24(12):10184.
23. Kong Ho S, Leu HB, Wu CC, Yeh HI, Yin WH, Lin TH, et al. The prognostic significance of the presence of tenascin-C in patients with stable coronary heart disease. Clin Chim Acta. 2022; 535:68-74.
24. Závada J, Uher M, Svobodová R, Olejárová M, Hušáková M, Ciferská H, et al. Serum tenascin-C discriminates patients with active SLE from inactive patients and healthy controls and predicts the need to escalate immunosuppressive therapy: a cohort study. Arthritis Res Ther. 2015;17(1):341.
25. Hasegawa M, Yoshida T, Sudo A. Tenascin-C in Osteoarthritis and Rheumatoid Arthritis. Front Immunol. 2020;11.
26. Ning L, Li S, Gao J, Ding L, Wang C, Chen W, Shan G, Zhang F, Yu J, Xu G. Tenascin-C Is Increased in Inflammatory Bowel Disease and Is Associated with response to Infliximab Therapy. Biomed Res Int. 2019; 2019:1475705.
27. Page TH, Charles PJ, Piccinini AM, Nicolaidou V, Taylor PC, Midwood KS. Raised circulating tenascin-C in rheumatoid arthritis. Arthritis Res Ther. 2012; 14(6):R260.

Disclaimer/Publisher's Note: The statements, opinions and data contained in all publications are solely those of the individual author(s) and contributor(s) and not of MDPI and/or the editor(s). MDPI and/or the editor(s) disclaim responsibility for any injury to people or property resulting from any ideas, methods, instructions or products referred to in the content.
